# Multivesicular Liposome (Depofoam) in Human Diseases

**DOI:** 10.22037/ijpr.2020.112291.13663

**Published:** 2020

**Authors:** Bahare Salehi, Abhay P. Mishra, Manisha Nigam, Farzad Kobarfard, Zeeshan Javed, Sadegh Rajabi, Khushbukhat Khan, Hafiz Ahsan Ashfaq, Toqeer Ahmad, Raffaele Pezzani, Karina Ramírez-Alarcón, Miquel Martorell, William C. Cho, Seyed Abdulmajid Ayatollahi, Javad Sharifi-Rad

**Affiliations:** a *Noncommunicable Diseases Research Center, Bam University of Medical Sciences, Bam, Iran. *; b *Student Research Committee, School of Medicine, Bam University of Medical Sciences, Bam, Iran. *; c *Department of Pharmaceutical Chemistry, H. N. B. Garhwal (A Central) University, Srinagar Garhwal, 246174, Uttarakhand, India. *; d *Department of Biochemistry, H. N. B. Garhwal (A Central) University, Srinagar Garhwal, 246174, Uttarakhand, India. *; e *Phytochemistry Research Center, Shahid Beheshti University of Medical Sciences, Tehran, Iran.*; f *Department of Medicinal Chemistry, School of Pharmacy, Shahid Beheshti University of Medical Sciences, Tehran, Iran. *; g *Office for Research innovation and commercialization (ORIC) Lahore garrison University, sector-c phase VI, DHA, Lahore Pakistan.*; h *Department of Clinical Biochemistry, School of Medicine, Shahid Beheshti University of Medical Sciences, Tehran, Iran.*; i *Atta-ur-Rahman School of Applied Biosciences (ASAB), National University of Sciences and Technology (NUST), Islamabad 44000, Pakistan. *; j *OU Endocrinology, Dept. Medicine (DIMED), University of Padova, via Ospedale 105, Padova 35128, Italy. *; k *AIROB, Associazione Italiana per la Ricerca Oncologica di Base, Padova, Italy. *; l *Department of Nutrition and Dietetics, Faculty of Pharmacy, and Centre for Healthy Living, University of Concepcion, Concepcion 4070386, Chile. *; m *Universidad de Concepción, Unidad de Desarrollo Tecnológico, UDT, Concepcion 4070386, Chile.*; n *Department of Clinical Oncology, Queen Elizabeth Hospital, 30 Gascoigne Road, Hong Kong, China. *; o *Department of Pharmacognosy and Biotechnology, School of Pharmacy, Shahid Beheshti University of Medical Sciences, Tehran, Iran.*

**Keywords:** DepoFoam, Depocyt, Encapsulation, Nanotechnology, Drugs

## Abstract

Drug development is a key point in the research of new therapeutic treatments for increasing maximum drug loading and prolonged drug effect. Encapsulation of drugs into multivesicular liposomes (DepoFoam) is a nanotechnology that allow delivery of the active constituent at a sufficient concentration during the entire treatment period. This guarantees the reduction of drug administration frequency, a very important factor in a prolonged treatment. Currently, diverse DepoFoam drugs are approved for clinical use against neurological diseases and for post-surgical pain management while other are under development for reducing surgical bleeding and for post-surgical analgesia. Also, on pre-clinical trials on cancer DepoFoam can improve bioavailability and stability of the drug molecules minimizing side effects by site-specific targeted delivery. In the current work, available literature on structure, preparation and pharmacokinetics of DepoFoam are reviewed. Moreover, we investigated approved DepoFoam formulations and preclinical studies with this nanotechnology.

## Introduction

The most challenging issue in drug development is designing appropriate delivery system. While designing sustained released formulation for high molecular weight drugs, scientists’ major concern is to achieve maximum drug loading for prolonged therapeutic effect (1). Microparticulate system technology which exploits the characteristics of multivesicular liposome has provided a pragmatic solution to this challenge. Its multi-compartment structure allows the retention of drug for longer time. This release-extension also eliminates the requirement for continuous in-take of drug (2, 3). 

**Figure 1:**
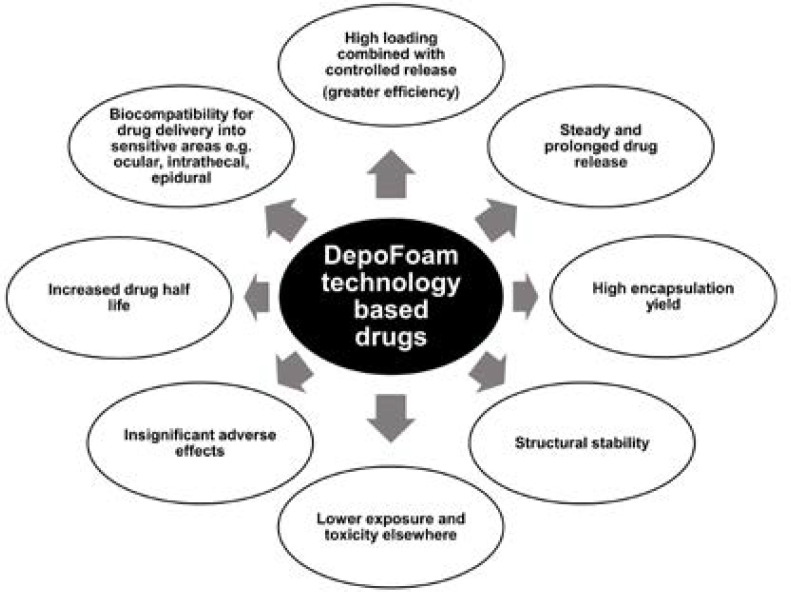
Advantages of multivesicular particles (DepoFoam) based drugs

DepoFoam technology, apart for prolonging therapeutic treatment and reducing administration frequency (3), has an additional benefit cancelling toxic reaction (Figure 1).

As these particles are synthetic cognates of naturally occurring lipids in human bodies, on delivery they fully disintegrate without inducing inflammation (4, 5).

Currently, 3 DepoFoam drugs (DepoCyt, DepoDur and EXPAREL) are approved by FDA (U.S. Food and Drug Administration) and are successfully utilized against neurological diseases and for post-surgical pain management (6, 7). Two more DepoFoam drugs are under development including DepoTXA (DepoTranexamic Acid) for reducing surgical bleeding and DepoMLX (DepoMeloxicam) for post-surgical analgesia (8).

*Structure*

The healing effect of a drug strongly relies on its time in circulation. Intravenous or subcutaneous administration of therapeutic peptides or proteins often leads to their rapid clearance from the circulation. Their rapid clearance brings about the need for frequent injection of drug to maintain the required therapeutic levels. The use of multivesicular particles as alternative drug delivery system for the sustained release of macromolecular drugs in this regards has proven to be promising (4).

**Figure 2:**
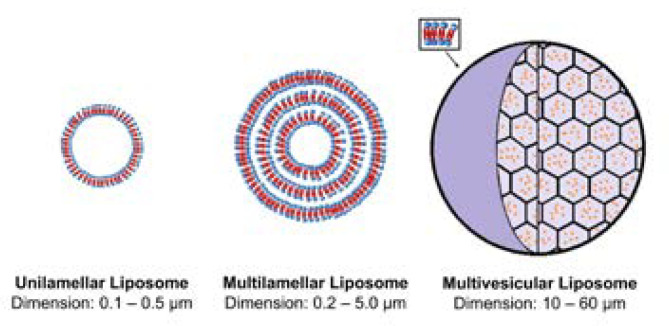
Main types of liposomes: unilamellar liposome, multilamellar liposome and multivesicular liposome

DepoFoam technology makes use of multivesicular liposomes (MVLs) particles, which appear as spheroids with granular structure under light microscope. Its average diameter is 10-12 μm. The architectural structure of MVLs bears the likeliness of aggregated soap bubbles as they consist of hundreds of polyhedral aqueous compartments where each compartment is separated by septa (Fig 2). According to a study in which confocal microscopy and fluorescent probes are used, the septa are composed of lipid bi-layer in which the outer layer is built up of phospholipids while the septa intersections are made up of triglycerides. The discrete presence of triglycerides is of chief importance as they act as membrane stabilizers and without these septal anchors, MVLs would be unstable (9-12). The stability of MVLs is also associated with the distribution and shape of polyhedral aqueous compartments. The arrangement of these compartments is in agreement with the poly-tetrahedral close packing which makes MVL a meta-stable structure (9, 13).

Proprietary data and analyses claim that all the components of DepoFoam are synthetically analogous to naturally occurring lipids which make them biologically compatible and degradable. These claims are also proven by the study in rat which reported the breakdown of DepoFoam’s predominant lipid component by regular metabolic pathways (14). Few recent studies have reported the side-effect and safety profile of DepoFoam drugs (15, 16) associated with its metabolites produced. In a safety evaluation of EXPAREL administered by repeated subcutaneous injection in rabbit and dogs, it has been reported that there was no indication of local or systemic complications (15). In addition, in a retrospective study pooling 6 prospective clinical trials that used liposome bupivacaine (EXPAREL) suggests that this formulation has a similar safety and side effect profile to bupivacaine HCl and placebo (normal saline), suggesting that most of the more common adverse effects are related to either opioid rescue or the surgical procedure itself (16). 

A list of various multivesicular particles (DepoFoam) drugs indications are shown in Table 1.

*Technology*

*Preparation method of multivesicular particles*

Although there are different methods for preparing the multivesicular particles, all approaches use very similar ways to produce these liposomal carriers (17-20). The preparation of multivesicular particles involve a double emulsification process which consists of two steps to form a water-in-oil-in-water emulsion. During the first step, a “water-in-oil” emulsion is formed. To prepare this emulsion, different lipid compositions including cholesterol, triglyceride, phospholipids, triolein or tricaprylin are solved in chloroform. Afterwards, this solution is further emulsified with an equal volume of an aqueous solution containing drug of interest and different amounts of sucrose that helps to prepare water-in-oil emulsion. The first emulsion is then emulsified with a second aqueous solution containing L-lysine, glycine or glucose to produce the water-in-oil-in-water emulsion. Chloroform is removed by flushing nitrogen over the surface of the prepared emulsion at 37 °C, to form the multivesicular particles. Finally, to remove unencapsulated drug, the resulting liposome particles are washed, and then harvested by centrifugation for 10 min at 600×g. Furthermore, MVLs can be dispersed in buffered saline solution. To assess the stability of the multivesicular particles matrix, the concentration of free drug in the supernatant is determined, which indicates the concentration of the drug released subsequent to the wash steps (18). 

*Pharmacokinetics of multivesicular particles*

Pharmacokinetic features of different multivesicular particles formulations have been investigated in several animal and human studies (17, 21, 22). Multivesicular particles have been administered by multiple routes including intrathecal, epidural, intraocular, intraperitoneal, subcutaneous, and intramuscular (23, 24). Depending on drug formulation, dose of administration, and way of injection, this technology can lead to increase drug half-life up to 600 times (24). Pharmacokinetic properties of most representative examples of DepoFoam-encapsulated drugs are discussed below. 

Zhao Y, Liu J, Sun X, Zhang Z-R and Gong T (22) studied hydroxycamptothecin (HCPT) both free and DepoFoam-encapsulated. They showed that the plasma levels of free HCPT was initially high after the administration of solution to the rat models and decreased immediately. Differently the concentration of DepoFoam-encapsulated HCPT retained steadily after initial burst release. In addition, the drug was detectable over a period of 6 days. They also reported that mean residence time (MRT) and t_1/2 _for DepoFoam-HCPT increased by a factor of 30-50 (22). Results from a different study conducted to design a repository system for delivery of ropivacaine hydrochloride through MVLs (RP-MVLs) indicated that t_1/2_, MRT and the area under the concentration–time curve (AUC) were remarkably increased when the drug was encapsulated in DepoFoam (21). In addition, the time to reach maximum (T_max_) was significantly higher than free drug indicating the sustained release of drug by multivesicular particles. The authors also suggested that the large size of encapsulated drug could help escaping from the absorption of the lymph or capillary network. Moreover, multivesicular particles has multiple aqueous chambers with drugs inside that could play a role as a drug-depot (21). 

*Approved multivesicular particles (DepoFoam) formulations*

As above mentioned, the most reported routes for multivesicular particles drugs administration are endovenous (intravenous), subcutaneous, intrathecal, epidural, and intraocular. The effectiveness of each route depends on the nature of the drug and underlying disease (Table 1). 

*DepoCyt*

DepoCyt® (also known as DTC 101), a liposomal product containing cytarabine/Ara-C is a pyrogen-free, parenteral suspension of the antimetabolite Ara-C, developed for neoplastic meningitis (NM) treatment by controlled release of Ara-C developed by encapsulating the aqueous drug solution in multivesicular particles (DepoFoam) (4, 25, 26). Cytarabine (cytosine arabinoside, Ara-C), an analogue of the nucleoside’s cytidine and deoxycytidine, contains arabinose rather than ribose or deoxyribose. It is an S-phase–specific drug, whose prolonged exposure of the cells to cytotoxic concentrations is critical to achieve maximum cytotoxic activity. It was approved for the treatment of lymphomatous meningitis by the FDA in 2000 (27). Due to intrathecal delivery, drug persists in cerebrospinal fluid (CSF) and allows complete exposure of cytarabine to tumor cells (28-30). Also, due to negligible systemic exposure, lower doses of cytarabine are effective as compared to drug administered systematically (31). Additionally, cytarabine is teratogenic in nature and its intrathecal administration in gestating patients minimizes the risk of fetal damage (29). Common side effects of DepoCyt include headache, nausea, vomiting, and fever which later lead to a severe condition called arachnoiditis. Dexamethasone is co-administrated to avoid occurrence or severity of this condition (32). 

Clinical studies focusing on the pharmacokinetics, antitumor activity, and safety aspects of these MVLs encapsulating cytarabine were explored in the patients with NM secondary to various hematologic and non-hematologic malignancies. In a phase I clinical study, nine patients with leptomeningeal metastasis were treated with 1–7 cycles of DepoCyt (ranging from 25 to 125 mg) into the lateral ventricle via Ommaya reservoir (33). Five out of six patients, who were eligible for cytological evaluation for CSF reported CSF being cleared of malignant cells within 3 weeks after initial treatment (33). The same group of researchers further documented the improved pharmacokinetics of cytarabine in the form of a DepoCyt injection (34). 12 patients received an increasing dose of DepoCyt via single intraventricular (IVT) injection or lumbar intrathecal injection (2–3 weeks for 15– 27 cycles). Therapeutic ventricular CSF concentration of cytarabine was found to be maintained for 9 ± 2 days after the IVT injection whereas therapeutic intralumbar concentration of cytarabine was maintained for up to 14 days after the intralumbar injection, with 7 out of 9 patients receiving DepoCyt via this route exhibiting some cytological responses with maximum tolerated dose of 75 mg. Although both routes managed to maintain cytarabine concentrations above the minimal cytotoxic level for about 14 days, an advantage for intralumbar administration was observed since it provoked higher cytarabine CSF concentrations (34). To examine the efficacy and safety of DepoCyt in comparison with conventional cytarabine a randomized trial of 28 patients with lymphomatous meningitis was conducted (35). It was documented that DepoCyt injection was more effective than a conventional 50 mg injection of cytarabine every 2 weeks, as evident by the significantly better elimination of malignant cells from CSF in DepoCyt-treated patients (71%) as compared to conventional cytarabine (15%). However, Depocyt treatment was frequently associated with transient symptoms of arachnoiditis, such as headache and nausea/vomiting (35). A multi-centre cohort study was performed to evaluate safety and efficacy of DepoCyte for intrathecal treatment of NM secondary to breast cancer. 51 patients were injected 50 mg of DepoCyt once every two weeks for 1 month of induction therapy followed by an additional 3 months of therapy if responded to the treatment (36). Of 43 patients 28% responded whereas the median time to neurologic progression was 49 days and median survival time was 88 days. In the second study, 110 patients diagnosed with NM were given the same treatment by either lumbar puncture (LP) or IVT injection (37). Similarly, out of 70 patients cytologically evaluated 27% showed some responses (37). Adverse events were headache and arachnoiditis throughout both studies (36, 37). 

DepoCyt treatment was also previously compared with methotrexate, in the patients with NM secondary to solid tumours (38). 61 patients with histologically proven cancer and positive CSF cytologies were randomized, 31 patients received intrathecal DepoCyt (up to six 50-mg doses over 3 months) and 30 patients received methotrexate (10-mg doses over 3 months). 26% patients receiving DepoCyt and 20% patients receiving methotrexate, demonstrated some responses. In the DepoCyt-treated patients the median time to neurological progression was 58 days, and in methotrexate-treated patients it was 30 days. Median survival time was also found to be higher for the patients receiving DepoCyt (348 versus 98 days). Results concluded that DepoCyt treatment produced a response rate comparable to that of methotrexate, and significantly improved the time to neurological progression while offering the benefit of a less demanding dose schedule.

To determine the maximum-tolerated dose, the dose-limiting toxicities, plasma and CSF pharmacokinetics, the Phase I trial of intrathecal DepoCyt was performed in 18 children aged 3–21 years with advanced meningeal malignancies (39). The patients with advanced NM secondary to leukaemia or solid CNS tumours received Depocyt ranging from 25 to 50mg every 2 weeks during induction, once every 4 weeks during consolidation, and once every 8 weeks during the maintenance phase of treatment. Arachnoiditis-associated symptoms comprising headache, vomiting and nausea were evident at 25 mg dose. The maximum tolerated dose and recommended phase II dose was reported to be to 35 mg, administered with dexamethasone (0.15 mg/kg, twice a day for 5 days).

*DepoDur*

Multivesicular particles technology is also being used to tackle pain management after surgery. Pain control after surgery is one of the major concerns of physicians around the globe. Improper pain relief not only causes unnecessary discomfort but is also associated with chronic pain development, delayed hospital discharge and increased probability of postoperative complications such as pneumonia or myocardial infarction, etc. Generally morphine is used to treat post-surgery pain; however, it depletes from human system within 24 h and also raises the risk of central nervous system (CNS) infection and epidural hematoma formation, when epidurally administered (4). 

DepoDur (previously known as DepoMorphine) is a drug based on multivesicular particles technology approved for clinical use. It is a morphine sulfate extended-release liposome injection, product of Pacira Pharmaceuticals approved by FDA in 2004 as a post-surgical pain reliever. In a randomized, dose-ranging study to evaluate and compare the analgesic efficacy of a novel single-dose extended-release epidural morphine (EREM, Depodur) with standard epidural morphine in patients undergoing lower abdominal surgery, it was found that the subjects receiving EREM reported significantly lower pain-intensity scores and greater satisfaction with their pain relief. Moreover, the side effect profile of single-dose EREM was acceptable and predictable with 97% of adverse events rated as mild to moderate and consistent with those of other epidural opioids (40). EREM provides superior and prolonged post-cesarean analgesia compared to conventional epidural morphine with no significant increases in adverse events (41).

*DepoDFO*

Another drug, desferrioxamine mesylate (DFO) which is an excellent iron chelator, faces a problem of sort. As it is poorly absorbed through gastrointestinal track (GIT) and having a short half-life, it is parentally administered periodically (after every 8-12 h) (42). Toliyat T, Jorjani M and Khorasanirad Z (43) encapsulated DFO (DepoDFO) and reported that in comparison to Desferal (unencapsulated DFO), DepoDFO induced the urinary excretion of iron in rats 3 times greater, after 24 h of subcutaneous delivery of the first dose (43). Moreover, the total amount of DepoDFO released after 9 days is 57%, suggesting its extended release and eliminating the need for frequent doses.

*EXPAREL*

DepoFoam bupivacaine or EXPAREL is another analgesic used before and after surgical procedure. Usually it is infused subcutaneously at the site of surgery and can be taken orally. A recent study by Day KM, Nair NM, Griner D and Sargent LA (6) reported the comparison of oral and subcutaneous administration of EXPAREL and free bupivacaine in children after pharyngoplasty. According to the study, the patients who took EXPAREL whether subcutaneously or orally, showed better and extended pain control along with decrease in opioid intake. Additionally, the drug enhanced chances for better recovery and reduced the days of hospitalization by approximately one day. Moreover, two years safety outcomes in the patients received EXPAREL during two breast augmentation studies showed no safety concerns that would negatively affect the integrity of the breast implants, even up to two years after the procedure (44). Recently, the toxicity analysis in dogs reported that the animals tolerated higher doses of EXPAREL as compared to free bupivacaine-HCl and displayed negligible adverse effects (45). The administration of EXPAREL through intravascular, intrathecal, and epidural route is comparatively safe, though transient adverse reaction such as decreased muscle tone or convulsions have been reported to its intravascular administration. EXPAREL was initially approved by FDA for use as a local anaesthetic by wound infiltration (46), but FDA recently expanded the approved use of this formulation to transversus abdominis plane (TAP) blocks (47). 

*DepoIGF-1*

The sustained release property of multivesicular particles has been also applied in growth factors category. Indeed, Katre NV, Asherman J, Schaefer H and Hora M (17) revealed that the free form of insulin-like growth factor I (IGF-I) disappeared from bloodstream in 1 day, but the release of this growth factor from multivesicular particles -IGF-I was sustained over a period of 5-7 days. They also found that the MRT and t_1/2_ of multivesicular particles -IGF-I have been increased by factors of 10 and 6 respectively, as compared with the free form of protein (17). 

*Preclinical experiments with multivesicular particles technology*

*General diseases (excluding cancer)*

The drugs which have low oral bioavailability or are not orally well-tolerated are mostly chosen to be delivered subcutaneously (49), as happens with hormones or dietary supplements. For example, liraglutide is a glucagon-like peptide-1 (GLP-1) agonist clinically approved in USA and Europe to treat diabetes mellitus (DM). Despite being the latest and the most advanced drug to treat this chronic disease, its frequent subcutaneous daily doses are required for lifetime. So, Zhang L, Ding L, Tang C, Li Y and Yang L (50) encapsulated it in lipid bi-layer with intention to enhance its plasma half-life after single dose and to reduce the frequency of doses. They reported that liraglutide-loaded MVLs (lrg-MVLs) retained its glucose-lowering activity even after 144 h while free liraglutide activity diminished within 30 h. 

Another study showed that the encapsulation of Leridistim (a protein from the myelopoietins family, as cytokines) in different formulations of multivesicular particles has been reported to enhance not only the neutrophil counts for 10 days, as compared to the free drug, but also could prolong the duration of live neutrophils from 2–3 to 9–10 days (19).

Grayson LS, Hansbrough JF, Zapata-Sirvent RL, Kim T and Kim S (51) encapsulated gentamicin (GENT) in multivesicular particles and incubated in human plasma with half-life of 21 days, demonstrating *in-vitro* stability. *In-vivo *pharmacokinetics revealed significant difference between GENT levels in tissue achieved by encapsulated drug or free drug. After 24 h from administration of free drug, there was a minimal detectable GENT level in tissues, while therapeutic levels of GENT remained constant in tissue at 24 h following multivesicular particles -GENT injection. Other mice received subcutaneous pretreatment on the dorsum with multivesicular particles -GENT or free GENT followed 2 days later with inoculation of 10^7^ *Staphylococcus aureus *in the site where the antibiotic had been injected previously. At 48 h post infection, colony-forming units (CFU) of *S. aureus *per gram of tissue were determined. The animals which had received multivesicular particles -GENT had a fourfold log_10_ reduction in CFU compared to the animals which received free GENT, sterile water, and non-drug containing multivesicular particles. Therefore, multivesicular particles drug delivery offers a promising method of sustained, high concentrations of antibiotic to local tissues while avoiding prolonged exposure to toxic systemic levels. 

In a study conducted by Qiu J, Wei XH, Geng F, Liu R, Zhang JW and Xu YH (52), an optimized and sustained release of MVL formulation of interferon (IFN) α-2b in female Sprague Dawley rat model was developed for the treatment of viral infections requiring less frequent dosing. It was reported that after subcutaneous injection, the MVL slowly released IFN α-2b into systemic circulation in a sustained manner with the estimated serum half-life of IFN α-2b was approximately 30 h. 

The efficacy of multivesicular particles -encapsulated amikacin sulfate (DEAS), a biodegradable a locally injectable antibiotic, was studied utilizing a foreign bodies infection mouse model. This model was applied with the aim to slow-release antibiotic formulation directly into infection sites so as to maintain therapeutic local drug concentrations and avoid systemic exposure to potentially toxic agents. Maintenance of local bactericidal concentrations for a prolonged period resulted in improved treatment compared with repeated systemic or local application of the free antibiotic thus hinting its clinical utility as locally injected antibiotic in certain infections (53). 

Insulin delivery is one of the issues on which efforts are centered. Apart from the side effects such as poor management of blood glucose level associated with conventional insulin therapy, its limitations further include thickening of capillary basement walls, nephropathy, retinopathy, and cardiovascular complications (54, 55). Further, due to rapid elimination rate, the bioavailability of the drug remains low which leads to shorter duration of drug contact with target site. Consequently to address these issues, an attempt was made to administer mucoadhesive MVL through intranasal and intraocular routes in rats (56). Within 8 h, mucoadhesive MVLs (i.e. chitosan and carbopol coated MVL) administered through intranasal route cleared 65% and 55% of blood glucose, respectively. These results were promising if compared to non-coated MVLs and conventional liposomes, which only reduced blood glucose by 32% in 12 h and 34% in 8 h, respectively. 

In addition, it has been shown that chitosan coated MVL administered through intraocular route showed better activity than carbopol coated MVL, with 30% reduction of blood glucose (56, 57).

Besides these, multivesicular particles (Depofoam) technology has also revolutionized the field of cancer therapy and the comprehensive information related to multivesicular particles -based cancer

*Cancer*

The genetic and phenotypic complexity of the cancer cells leads to the clinical diversity and therapeutic resistance, a major hurdle in the therapy of cancer. Chemotherapy, despite being one of the most common approach for cancer treatment, possesses critical limitations, such as poor bioavailability and severe side effects (58-61). Multivesicular particles (Depofoam) technology have revolutionized the concept of cancer therapy by overcoming these limitations via improving bioavailability and stability of the drug molecules and minimizing side effects by site-specific targeted delivery of the drugs (4). There are various liposomal formulations approved for cancer therapy such as Doxil® (PEGylated liposome), DaunoXome® (daunorubicin citrate liposomal formulation), Depocyt® (multivesicular liposome), Myocet® (nonpegylated liposomal formulation), Mepact® (multilamellar liposomes), Marqibo® (vincristine sulfate liposomal injection), and Onivyde™ (irinotecan liposome injection). As already mentioned, only Depocyt, a multivesicular liposome based formulation using DepoFoam technology, is approved by the FDA for clinical use in cancer therapy.

Prolonged maintenance of a therapeutic drug concentration in the CSF is required for optimal treatment of leptomeningeal leukemia or carcinomatosis with cell cycle-specific antimetabolites such as l-b-D-arabinofuranosylcytosine (Ara-C). Indeed, its half-life in humans is short and its frequent or continuous intrathecal administration is impractical through the intralumbar route. To meet this requirement Ara-C encapsulated into DepoFoam (Depo/Ara-C) was developed and studied, as above mentioned in paragraph n° 4.1. 

A study was performed to analyze the sustained release of HCPT, a DNA topoisomerase I inhibitor (cytotoxic antitumour compound derived from camptothecin), after subcutaneous administration using a novel phospholipid complex multivesicular particles technology (22). Preparation, characterization, *in-vitro* release, and *in-vivo* pharmacokinetics of HCPT–phospholipid complex-loaded MVLs (HCPT-MVLs) were investigated and it was documented that HCPT-MVLs could improve pharmacokinetic behaviours of the original drug, with a sustained release over 5–6 days (22).

Methotrexate, another drug used for cancer therapy and as immunosuppressant, was encapsulated into a lipid-based drug-delivery system to create a slow-release formulation (Depo/methotrexate) for intracavitary administration. Multivesicular particles methotrexate loaded was stable in storage at 4 °C for >4 months and the half-life of drug release was 40 days in human plasma. It was reported that after intraperitoneal injection of this formulation in mice, the apparent half-life of free methotrexate was 39.6 h, in contrast to a half-life of 0.5 h for the unencapsulated standard methotrexate. In murine leukemia model (L1210), the potency of a single dose of Depo/methotrexate was 334-fold higher, the increased life-span was 2-fold greater, and the therapeutic index was 2-fold higher than a single dose of standard methotrexate (62). Moreover, these studies were extended for subcutaneous administration in BDF1 mice too. It was reported that Depo/methotrexate increased the methotrexate plasma half-life from 0.53 to 100 h. Plasma peak levels of the encapsulated drug were 120-fold lower than those of unencapsulated methotrexate. Due to the extended drug release, the single-dose potency of methotrexate against the L1210 leukemia model increased by a factor of about 130 without changing the therapeutic index (63).

The use of MVLs as a slow-release depot of bleomycin, an antitumor antibiotic for systemic administration was assessed via the subcutaneous route against the B-16 melanoma model in BDF1 mice. It was documented that the therapeutic index of single-dose bleomycin subcutaneously given was significantly improved in the encapsulated drug in MVLs: the better efficacy was evident by inhibition of tumor growth, increased animal life span and the decreased toxicity (64).

Cisplatin, a platinum coordination complex with antineoplastic activity in many tumors, was encapsulated into MVLs and its pharmacokinetics, tissue distribution, and therapeutic efficacy were studied after subcutaneous injection of encapsulated and unencapsulated drug at the site of an experimentally induced tumor in mice. Cisplatin-MVLs were capable of high drug loading (0.148:1 mg cisplatin/mg lipid) and high encapsulation efficiency (>80%). An* in vitro* study showed the sustained release of encapsulated drug for >7 days with increased drug accumulation in liver, spleen, and tumor regions than free cisplatin solution. Also, the cisplatin-MVL therapeutic efficacy was significantly higher than that of cisplatin solution against S180 tumor-bearing mice (65).

A novel liposome using the antimetabolite 5-fluorouridine-5’-monophosphate (FUMP), a potent metabolite of 5-fluorouracil was developed for sustained drug delivery to the eye. The *in-vitro* half-life of FUMP-liposomes was 585 h. Subconjunctival administration of 1 mg of FUMP to white rabbits, documented the significantly greater tissue drug level at the injection site in the group treated with liposomes as compared to unencapsulated FUMP (66). Moreover, the effects of the antimetabolites, cytarabine (Ara-C), and FUMP, encapsulated in MVLs on suppression of proliferative vitreoretinopathy in New Zealand white rabbits were studied. Pharmacokinetic studies revealed the extended 124 h half-life of the drug in the vitreous cavity in contrast to 4.5 h of nonliposome-treated controls after intravitreal administration. In a heterologous dermal fibroblast model of proliferative vitreoretinopathy, there was a 92% decrease in frequency of tractional retinal detachments in the rabbits, receiving a single intravitreal injection of liposome-encapsulated 0.1 mg of FUMP compared with the controls receiving liposomes without drug. Whereas, under similar conditions, Ara-C was significantly less effective than FUMP, providing only a 46% reduction in tractional membranes (67). 

*-*

These studies provide clear evidence that encapsulation of several antineoplastic agents consistently resulted in the sustained release of these drugs. The drugs were injected by various routes and their concentrations were determined in plasma, CSF, vitreal fluid, and different tissue. Encapsulation of drug unequivocally prolonged the half-life. Many works reported the positive effects of encapsulation with DepoFoam technology, but up to now very few compounds entered the clinical practice. It is clearly apparent that the technology of multivesicular particles (DepoFoam) delivery system largely enhances the bioavailability and pharmacodynamics of delivered drugs or factors by improving the half-life and MRT of them. These prominent characteristics of this technology could aid in decreasing the limitations and toxic effects of conventional therapies. Given the great potential of this technology it is necessary to expand our knowledge on DepoFoam and also further efforts should be rapidly profuse to implement its use in human diseases.

**Figure 3:**
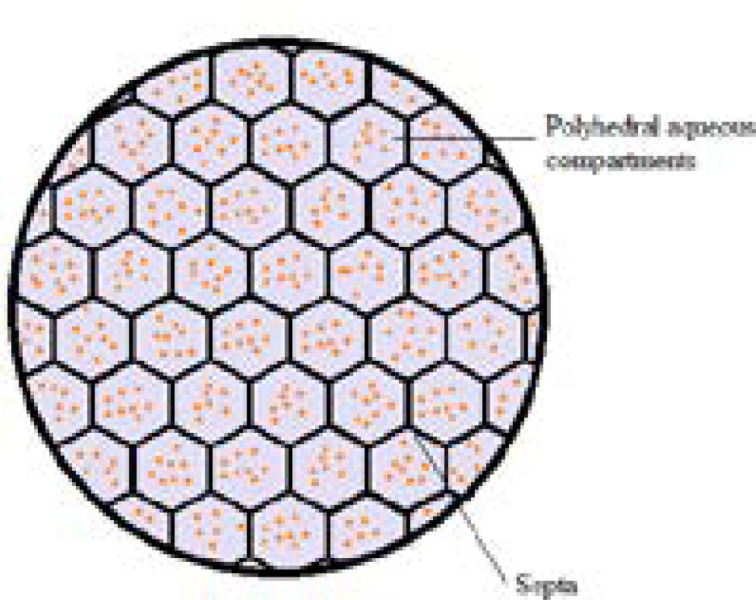
Schematic representation of multivesicular liposome (MVL). Hundreds of discontinuous polyhedral aqueous chambers are separated one each other by the continuous network of lipid bi-layer septa

**Table 1:** Multivesicular particles (DepoFoam) drugs indications

**Formulation**	**Drug**	**Indication**
DaunoXome	Daunorubicin	Kaposi's sarcoma
DepoCyt	Cytabarine	Neoplastic meningitis and lymphomatous meningitis
DepoDFO	Desferrioxamine mesylate	Iron chelator
DepoDur	Morphine sulfate	Postoperative pain following surgery
DepoIGF-I	insulin-like growth factor I	Hormonal disorder
DepoMLX	Meloxicam	Postoperative pain following surgery
DepoTXA	Tranexamic acid	Surgical blood loss
Doxil	Doxorubicin	Refractory Kaposi's sarcoma, recurrent breast cancer and ovarian cancer
EXPAREL	Bupivacaine	Postoperative pain following surgery
Marqibo	Vincristine sulfate	Acute lymphocytic leukemia
Myocet	Doxorubicin	Recurrent breast cancer
Onivyde	Irinotecan	Cancer treatment

**Table 2:** List of multivesicular particles (DepoFoam) drugs along with their half-life and route of administration

**DepoFoam**		**Against**	**Subject**	**Administration**	**Half-life (h)**		**Reference**
**Drug**	**Active substance**				**Encapsulated**	**Unencapsulated**	
DepoCyt	Cytarabine liposome	Lymphomatous meningitis	Human	Intrathecal	82.4	3.4	(29)
DepoDur	Morphine	Pain	Human	Epidural	24-48	12-24	(2)
DepoDFO	Desferrioxamine mesylate	Iron overload	Rat	Subcutaneous	72	24	(43)
EXPAREL	Liposomal Bupivacaine	Pain	Human	Subcutaneous	96	24	(3, 48)
			Dog	Intravenous	1.11	0.473	(45)
				Intra-arterial	0.539	0.26	
DepoIGF-I	Insulin-like growth factor I	Hormonal disorder	Rat	Subcutaneous	26	4	(17)
